# Improving the Pharmacists’ Response to Public Health Emergencies—Documentary Research on Online Resources Provided by National Pharmacists’ Associations

**DOI:** 10.3389/ijph.2022.1604537

**Published:** 2022-08-24

**Authors:** Alexandra Toma, Ofelia Crişan

**Affiliations:** Pharmaceutical Legislation and Management, Department IV Pharmacy, Faculty of Pharmacy, “Iuliu Haţieganu” University of Medicine and Pharmacy, Cluj-Napoca, Romania

**Keywords:** pharmacists, national pharmacists’ associations, COVID-19 pandemic, public health emergencies, preparedness and response, recommendations for training and communication

## Abstract

**Objectives:** Given the expanding role of pharmacists during COVID-19 pandemic, we aimed to investigate the approach of professional associations to supporting their practice and to find ways to improve their response to public health emergencies.

**Methods:** We conducted documentary research on websites of seven national pharmacists’ associations, submitted the findings to a comparative thematic analysis, and made proposals of specific good practices.

**Results:** Many great resources were provided by pharmacists’ associations in Australia, France, Spain, and the US. The similarities include scientific information on COVID-19 tests, treatments and vaccines, legal issues, and wellbeing management. The main differences were in developing medication management programs, supporting hospital pharmacists, helping families, or advocating for an equitable vaccination. In Finland, Hungary and Romania, the focus was on updating emerging information. Considering the need for better managing public health emergencies at organizational level, we suggested good practices regarding training and communication.

**Conclusion:** Professional associations should develop preparedness and response plans for public health emergencies. Practical training and effective communication could improve the resilience of pharmacists and patients during pandemics, which could save lives.

## Introduction

Since the beginning of the COVID-19 pandemic, pharmacists, along with other healthcare professionals, have been on the front lines fighting against this disease [1–3]. To address this public health emergency, first, pharmacists had to adapt their professional activities to prevent infection with SARS-CoV-2 virus [3–5]. Second, they began using long-distance communication services by implementing telepharmacy or telehealth activities to support patients or other healthcare professionals [5–8]. Additionally, in the context of the COVID-19 pandemic, in some countries, pharmacists have been granted the right to prescribe to facilitate the further treatment of patients with chronic disease [4, 6]. Furthermore, pharmacists were involved in testing for SARS-CoV-2 infection as well as in vaccination campaigns [4, 6, 9]. At the same time, pharmacists had to maintain their psychological and emotional well-being to manage the stress and pressure caused by the pandemic [10, 11].

Taking into consideration all the above, pharmacists needed specific information, training, and support [6, 9, 12–14]. To this end, at the international level, information hubs were created by professional organizations such as the International Pharmaceutical Federation (FIP) and Pharmaceutical Group of the European Union (PGEU) [1, 15]. Those hubs were used to share not only their own resources, but also several resources published by other organizations. For instance, FIP COVID-19 Information Hub contains guidance and examples of best practices from around the world, provided to pharmacists by member organizations and other institutions, to inspire them and their national associations to better deal with the fast-changing pandemic environment [16]. Developing new ways of communication and cooperation within and throughout such difficult circumstances is essential to secure patients’ continuous access to quality pharmaceutical care, while also ensuring workforce and environmental protection [17]. Therefore, coordinating efforts of pharmacists from all professional settings for a more “robust, sustainable response” and their full integration within public health and emergency preparedness and response systems would benefit to the future calls to duty [18].

FIP had already recommended the development of emergency preparedness plans for pharmaceutical actors to be better prepared to respond to natural disasters, such as disease outbreaks [19]. FIP also stated that pharmacists should accept their ethical role as first responders in such context, but they also need to be able to successfully manage all unexpected challenges during emerging health-related events in their communities [20]. At the national level, this kind of action falls under the task of pharmacists’ professional associations, and they are also responsible for monitoring practices for compliance with quality standards. As far as we know, there are no academic publications investigating the role of those associations in providing professional guidance and quality standards for their members’ activity during public health emergencies. Consequently, the objectives of this study were to investigate the approach of different national pharmacists’ associations to supporting their members’ practices during the COVID-19 pandemic, to analyse and compare specific resources and training programs provided by those associations through their official websites, and to propose good practices for improving their preparedness for and response to public health emergencies.

Because pharmacy good practices aim to provide quality services and improve the professional performance of pharmacists [21], we agreed to include countries based on the latest OECD assessments of the quality and performance of health systems, which also evaluated pharmacists as health service providers [22, 23]. According to OECD, the most used systems for covering health services are social health insurance systems and national health systems, but many countries combine those sources with “additional health coverage through voluntary private health insurance” [22]. Social health insurance systems, developed from the Bismarck model, cover health services through compulsory social security contributions paid by employers and employees and are adopted in many countries, such as Germany, France, Hungary, Poland, or Romania. National health systems, developed from the Beveridge model, cover health services through public taxes and are adopted also in many countries, such as the United Kingdom (UK), Spain, Italy, Finland, or Sweden. A third system, called the mixed model or private health insurance system, covers most health services through voluntary insurance schemes or upfront payments and is adopted in Austria, Bulgaria, or Greece [24]. Outside Europe, mixed systems were also adopted in Australia and the United States (US), including funding both from private health insurance and public contribution from states and the federal government, e.g., Medicare and Medicaid [22]. Pharmacists are reimbursed mostly for their services related to dispensing (through margins, maximum reimbursement price, fees), as in most European countries, such as Spain, Italy, Finland, Hungary, Poland, or Romania [25]. In some countries, they are reimbursed for providing clinical services (fees for administration of medicines, including vaccination, medicine use review, medication therapy management etc.), as in the UK, France, Australia, or the US [25–27].

## Methods

To gain depth in our research, the number of countries included in the study was decided based on the Small-N comparative analysis model, which involves the use of 3–10 systems to compare [28]. The selection of countries was designed to capture approaches from both similar and different health systems, looking from Eastern Europe to Western models. First, we included EU Member States with similar social health insurance systems covering health services: Romania, our country, and Hungary, for their common history and endeavours to improve healthcare quality in their systems [23] and France, as a country with one of the best social health insurance systems [22]. Second, we included two other advanced European countries with different health systems, covering health services through a decentralised national health system: Spain and a Scandinavian country, Finland, where “pharmacists also play an enhanced role in health promotion and disease prevention, including in rural areas” [22]. Third, we included two non-European countries, with mixed systems covering health services, classified by OECD as being among the most performant in the world: Australia, a country with very developed direct pharmaceutical services, and the US, where “pharmacists have been given greater scope including extending prescriptions, enabling electronic prescription transfer and, in some cases, prescribing medicines for certain chronic conditions” [22].

In each of those countries there is a national pharmacists’ association, representing all pharmacists and having authority over the entire professional body. We conducted documentary research [29] on the websites of the seven national pharmacists’ associations, as being functional equivalents, meaningfully comparable [28]. We searched for COVID-19 headlines, looking for specific resources addressing the pharmacists’ need for information, training, or support. We looked for equivalent resources in terms of content, to be comparable [28]. We collected existing and freely accessible online documents, submitted them to an iterative reading, analysing, and interpreting, then to a comparative thematic analysis [29]. We analysed those websites between September 2020 and September 2021, re-evaluating them monthly, to observe relevant changes in terms of resources of interest for our research. The analysis included the steps of exploring and understanding the documents, identifying themes, classifying them, and selecting those of main importance (commonly occurring themes during the progress of our research), then summarizing and comparing documents across countries [28, 29]. The comparison was based on three main criteria, crystalized during our research process: types of documents (in terms of origin, destination, and content), main professional themes covered and specific initiatives.

Considering the results of the comparison and following the recommendations of the National Academies of Sciences, Engineering, and Medicine, included in their report Evidence-Based Practice for Public Health Emergency Preparedness and Response [30], we engaged in a reiterative process of reflection and discussion, which led us to formulate proposals for specific good practices regarding training and communication, which professional associations might consider in order to improve their members’ resilience during pandemic situations.

## Results

The seven national pharmacists’ associations address the COVID-19 pandemic on their websites as follows:

- The Pharmaceutical Society of Australia has created a specific webpage named COVID-19 information for pharmacists [27];
- The Finnish Pharmaceutical Association does not have a specific webpage on the COVID-19 pandemic, but several documents on this matter can be found by searching on the front page/current affairs [31];
- The French National Chamber of Pharmacists has created on its website a specific webpage named COVID-19 [32], but many specific resources were developed by Cespharm, the health and social education committee of the French association [33];
- The Hungarian Chamber of Pharmacists does not have a specific webpage on the COVID-19 pandemic, but several documents on this matter can be found by searching the website [34];
- The Romanian College of Pharmacists does not have a specific webpage on the pandemic, only on the COVID-19 vaccines, but several documents on this matter can be found by searching the website [35];
- The General Pharmaceutical Council of Spain has created a specific webpage named SARS-CoV-2 (COVID-19) coronavirus infection [36];
- The American Pharmacists Association has created a specific webpage named Pharmacists’ guide to coronavirus [37].

Different types of documents were found on the analysed websites. Internal documents, created by the national pharmacists’ associations, some of them in collaboration with partners and/or competent authorities, and external documents, created by other institutions, were identified. Additionally, documents for both members and patients were published. A certain amount of variation in their content was found, including news about the pandemic evolution, documents providing support for public or patients, and resources for training and support for pharmacists. A comparative synthesis is presented in Table 1.

**TABLE 1:** Types of documents published by pharmacists’ associations (7 countries, 2020–2021).

In terms of		Australia	Finland	France	Hungary	Romania	Spain	United States
Origin	Internal	+	+	+	+	+	+	+
	External	+	−	+	+	+	+	−
Destination	Public	+	−	+	+	+	+	+
	Pharmacists	+	+	+	+	+	+	+
Content	News	+	+	+	+	+	+	+
	Support for patients/public	+	−	+	−	−	+	+
	Training/support for members	+	−	+	−	−	+	+

The main professional themes addressed in the documents published on the national pharmacists’ associations’ websites were pertaining to solving legal issues regarding extended prescription-related rights and reimbursements during the pandemic, protecting staff and patients from contamination and fatigue, improving pharmaceutical care for local communities, and developing new activities in pharmacy. A comparative synthesis is presented in Table 2.

**TABLE 2:** Professional themes covered by pharmacists’ associations (7 countries, 2020–2021).

Themes	Australia	Finland	France	Hungary	Romania	Spain	United States
Legal issues during COVID-19 pandemic	+	+	+	+	+	+	+
Protection from COVID-19	+	−	+	+	+	+	+
COVID-19 testing	+	−	+	−	+	+	+
COVID-19 treatments	+	−	+	+	+	+	+
COVID-19 vaccination	+	+	+	+	+	+	+
Helping communities	+	+	+	+	+	+	+
Telehealth/telepharmacy	+	−	+	−	+	−	+
Medication management	+	+	+	−	−	+	+
Wellbeing management	+	+	+	+	+	+	+
Pharmacy management	+	−	+	−	+	+	+

Each of the seven national pharmacists’ associations also had specific initiatives during the COVID-19 pandemic, addressing the most pressing professional issues: supporting research in the field, ensuring access to medication for vulnerable patients, dispensing patient education, providing specific guidelines for pharmacists working in different healthcare settings, and supporting pharmacists as frontline health professionals. A comparative synthesis of those initiatives is presented in Table 3.

**TABLE 3:** Specific initiatives of pharmacists’ associations (7 countries, 2020–2021).

Country	Specific initiative
Australia	Adapting medication management programs during COVID-19 pandemic and developing resources to help pharmacists providing services for vulnerable patients via telehealth arrangements [27, 38]
Finland	Cooperating within the Nordic Pharmaceutical Union for ensuring a quicker response to emergencies by investing in vaccine research, manufacturing expertise and supply [31, 39]
France	Supporting hospital pharmacists in better managing their workplace, activities, and health during COVID-19 pandemic [33, 40]
Hungary	Supporting academic research on the impact of COVID-19 pandemic on the pharmacists’ profession and mental health [34, 41]
Romania	Providing local guidelines for pharmacists on protecting themselves and counselling patients in community pharmacies during COVID-19 pandemic [35, 42]
Spain	Supporting patients in assembling first aid kits for children going back to school during the COVID-19 pandemic and managing the pandemic fatigue [36, 43]
US	Advocating for an equitable vaccination and for recognizing pharmacists as professionals capable to be engaged in administering COVID-19 tests and vaccines [37, 44]

An initiative for improving the national pharmacists’ associations’ approach to public health emergencies should involve preparedness planning for a better professional response and for supporting pharmacists to deal with such situations, both at the organizational and individual levels. A preparedness and response plan would provide internal guidance on the specific practices needed during a public health emergency. Among evidence-based practices for public health emergency preparedness and response, training practitioners and communicating with them [30] are especially important in the field of pharmacy. Our proposals of good practices for training and communication related to public health emergencies that could be considered by national pharmacists’ associations are shown in Table 4.

**TABLE 4:** Proposals of good public health emergency practices for pharmacists’ associations (Romania, 2021).

Recommended practice	Preparedness phase	Response phase
Training pharmacists for public health emergencies	Developing training programs with clear scope and objectives regarding:	Collaborating with authorities during public health emergencies by
	- mental health management	- notifying services that pharmacists might provide
	- disease testing management in pharmacies	- assuming responsibility for the pharmacists’ services
	- vaccination management in pharmacies	- organizing monitoring task groups to communicate and solve practice issues
	- medical/biological waste management	Supporting pharmacists in applying new competencies and behaviours to
	- patient counselling and medication management via non-traditional tools (e.g., telepharmacy, e-mail, smartphone apps) and at home	- better manage mental health
	Providing training programs to members:	- provide and administer tests and vaccines in pharmacies
	- at large scale within the organization (e.g., via internet, apps, flash drives)	- ensure appropriate medical/biological waste management
	- using various materials and available resources to facilitate participation	- provide quality services counselling and medication management, in particular to at-risk patients (e.g., chronic, elderly, isolated, poor)
	- with roles and responsibilities regarding learning and competencies assessment (e.g., teams, projects, coordinators)	
	Updating training programs based on:	
	- continuous feedback from members	
	- implementing new ideas and projects	
	- needs resulting from emerging circumstances	
Communicating with pharmacists during public health emergencies	Developing specific communication strategies regarding:	Collaborating with authorities during public health emergencies by:
	- internal information vetting and transmission	- notifying national and local persons of contact to facilitate communication
	- alerts and guidance from public authorities	- timely transmission of alerts and guidance to members
	Assigning communication channels depending on urgency—e.g	- communicating relevant feedback from members
	- website, intranet and/or e-mail for internal information	Supporting pharmacists by
	- text messages and/or smartphone apps for alerts and urgent guidance	- avoiding transmission of redundant, excessive, and contradictory information
	Encouraging participation based on	- providing information on and access to services of mental health for pharmacists and their teams
	- asking for and considering feedback	
	- establishing roles and responsibilities for members (e.g., coordinators, contact points)	
	- supporting local initiatives to develop communication with at-risk communities	

## Discussion

The seven national pharmacists’ associations’ websites show a great variety of resources and programs created during the COVID-19 pandemic. The studied national pharmacists’ associations from Australia, France, Spain, and the US developed by far the most easily accessible, information rich and interesting COVID-19 webpages. They provided useful content on many topics of interest for supporting their members and public through distinct and explicit informational and training materials using different communication tools, such as guidelines, posters, videos, or webinars. Those materials were complemented with several links to important resources on websites of other associations and health authorities, as is the case for the American Pharmacists Association. In contrast, in Finland, Hungary and Romania, the studied pharmacists’ associations were focused on keeping their members up to date with emerging information published regularly on their websites, concisely covering the relevant themes, particularly that of COVID-19 vaccines.

According to FIP and many researchers, COVID-19 vaccination campaigns would benefit from the addition of pharmacists [6, 9, 12, 45–47]. However, to be able to administer COVID-19 vaccines, pharmacists need training, especially in countries where pharmacists do not have such competency, such as Finland, Hungary, or Spain [3, 45, 46]. Even though all studied websites address the theme of COVID-19 vaccination, not all seven associations provided specific training programs through their websites. For example, in Romania, pharmacists’ right to administer vaccines was recently regulated, but they are not yet trained for this purpose. The Romanian College of Pharmacists should develop training programs on managing vaccination in pharmacies and learn from professional associations which are more advanced in vaccination, such as Australia, France, or the US [48]. Also, the Romanian College of Pharmacists and other interested professional associations should follow the recommendations to implement vaccination practices from the recently published FIP vaccination reference guide: Knowledge and skills to support professional development and inform pharmacy education in vaccination [49].

The studied pharmacists’ associations from Australia, France and the US also supported the implementation of telehealth or telepharmacy as new ways to ensure the accessibility and continuity of counselling and medication management for patients during the COVID-19 pandemic. Medication management via telehealth was developed and promoted by the Pharmaceutical Society of Australia to ensure the best services for vulnerable and home-isolated patients [38]. In Romania, the Romanian College of Pharmacists advised their members to use a free smartphone app, Pharmacist’s Recommendation, to help them coordinate with patients to monitor their biological parameters and treatments during the COVID-19 pandemic [42, 50]. Although telepharmacy is not particularly addressed by the Spanish pharmacists’ association, some studies emphasize that such services are developed and practised in Spain by hospital pharmacies to support their outpatients, in line with the position expressed by the Spanish Society of Hospital Pharmacy [8, 51].

Another association concerned with helping hospital pharmacists was the French National Chamber of Pharmacists, which developed guidelines for protecting the health of their teams and families through routines and barrier gestures [40]. Such guidelines are very important, as illustrated by FIP in the COVID-19 Information Hub, by giving the example of the guidance provided by the Chinese Pharmaceutical Association [16]. That guidance was meant to help the hospital pharmacists to actively participate in the COVID-19 treatment of hospitalized patients “to improve safety, efficacy and rationality of clinical drug use” and stay emotionally balanced at the same time [52]. A good preparation for dealing with public health emergencies, along with accurate communication to the people, would build up the framework for delivering quality health services and limit mental health threats, even in challenging circumstances of isolation, as testified in Malta [53]. Good guidance, including on maintaining mental balance, is of utmost importance for pharmacists because being able to maintain their own health is essential in providing help to others [10, 11]. The French association also published an impressive set of documents supporting vaccination in pharmacies (e.g., posters, procedures, checklists) developed by different authorities in France and attesting their cooperation with the pharmacists’ association in the interest of protecting public health [33].

As FIP recommended, public authorities from every country should recognize pharmacists as essential health professionals and involve and support them in managing the COVID-19 pandemic and developing pharmacy practice research [13, 47]. An interesting case of pharmacists’ participation in a multidisciplinary applied research team, exemplified by FIP in the COVID-19 Information Hub, was the project for finding an innovative COVID-19 treatment protocol, as response to pandemic in Africa [16, 54]. The extensive use of pharmacists’ knowledge and skills, from practice research to decision process on how to overcome ethical challenges and to ensure accessibility and continuity of healthcare during times of pressure, especially in lower-middle income countries, was further underlined as an urgency in the Commonwealth: “There needs to be ongoing support at the national and global level for pharmacy services to be implemented in their full potential” [17]. That same desideratum was identified in the US, where learning from experience and prepare to better respond would lead to “enhanced recognition of pharmacists’ skills, roles and contributions as integral members of the interprofessional healthcare team” [18]. Advocating for the recognition and valorisation of pharmacists’ competencies is a job for their professional bodies, which could follow the steps of the American Pharmacists Association in promoting the profession at all levels of authority [44]. Other initiatives of unity and solidarity within the profession, such as those of the Hungarian Chamber of Pharmacists and Finnish Pharmaceutical Association, even discreetly announced, deserve to be appreciated and emphasized as using their expertise to help the community [39, 41].

The most interesting materials for helping patients and families to stay mentally healthy and protected were published by the General Pharmaceutical Council of Spain, indicating an important concern for the wellbeing of local communities [43]. Additionally, the Spanish association could be regarded as a model in establishing good pharmacy practices for emergency situations, having already developed standard operating procedures covering several issues of interest (e.g., managing supplies, shortages, services in residential homes, alerts, health crises) that pharmacists have dealt with during the COVID-19 pandemic [55]. Into the same category of initiatives fall, as depicted by FIP in the COVID-19 Information Hub, the suggestions of the Canadian Pharmacists Association for best practices on the use of personal protective equipment (PPE) for pharmacy staff during the COVID-19 pandemic, denoting preoccupation for helping pharmacists appropriately use each piece of PPE, from when and how to use it to when and how to dispose of and/or decontaminate it [16, 56].

We admire the work and effort of national pharmacists’ associations, and we want to contribute to their endeavours through our proposals of good practices. We developed them based on the main professional themes they addressed and with the conviction that a more coherent approach to public health emergencies is needed at an organizational level. For pharmacists to better manage future public health emergencies it is necessary to improve their preparedness and response to such challenges, as stated by PGEU [15]. Well-articulated and implemented preparedness and response plans with culturally tailored training programs and communication strategies involving electronic messaging systems could improve competencies and behaviours and reduce morbidity and mortality [30].

Our proposals of good practices are in line with FIP guidelines for responding to disaster, which recommended to national organizations, during the response phase, to “identify mechanisms for appropriate means of communication to ensure delivery of timely information” with governments, pharmacists and public [19]. Also, our suggestions on encouraging and helping pharmacists to take the initiative in helping vulnerable communities fall into the scope of FIP statement addressing the pharmacists’ need to better manage disaster issues, as those arising during a disease outbreak [20]. Examples of similar approaches were further mentioned in the Commonwealth, including the use of new technologies, like apps and drones in West-African regions, as more innovative and flexible ways of delivering pharmaceutical care [17]. Furthermore, the pharmacists’ need for targeted training and education was underlined in the framework plan recommended in the US for coordinating those professionals with public health emergency preparedness and response efforts, to be able to strengthen them and establish new actions in the field [18].

As FIP stated, collaboration between science, research, education, and practice is essential “to set usable standards” in professional areas [57]. Therefore, like other peers who have devised proposals to improve the pharmacists’ preparedness and response to public health emergencies [13, 17, 18], we make the results of our research available to all practitioners and their professional associations. And we are ready to work with them to develop good practices for public health emergencies and train their members in implementing them. This way, pharmacists would be better prepared to respond to such situations, both as organizations and as individuals. This would result in positive outcomes for the patients and communities they serve, whose needs would be met by receiving all necessary preventive and curative services from their pharmacists.

### Limitations

First, this study is limited to seven countries due to our limited resources of time, but the main issues they covered have also preoccupied many other pharmacists’ associations during the pandemic, as shown above and from resources published by FIP from organizations around the world [16]. Second, from each chosen country, we included in our study the national pharmacists’ association, representing all pharmacists, and having authority over the entire professional body. We are aware that there are several other pharmaceutical organizations in each country, that are either employers’ or employees’ associations, with valuable contribution to the working environment, but with limited function and, therefore, not comparable [28] with the national associations. Third, we limited our documentary research to existing and freely online accessible resources [29] from the websites of national pharmacists’ associations, without reaching to them or their members for additional information, first, out of respect for their time, in a pandemic context, and second, because we thought those websites were rich enough to draw a relevant picture for their activities. Fourth, even if we are not familiar with the practice of all the exemplified countries, as pharmacists and scholars, we are able to evaluate and recognize the value of different resources for improving professional development and understand the importance and impact of having access to quality information, training, and support. Finally, the National Academies of Sciences, Engineering, and Medicine, in their report Evidence-Based Practice for Public Health Emergency Preparedness and Response, recommended implementing not only good practices of training and communicating with practitioners, but also activating public health emergency operation centers and implementing quarantine to reduce or stop the spread of a contagious disease [30]. We didn’t make proposals for good practices regarding those activities, because they do not fall within the remit of pharmacists’ associations.

### Conclusion

In the context of the COVID-19 pandemic, the role of pharmacists in providing healthcare has increased by expanding the field of activities carried out in pharmacies. The pharmacists’ associations were asked to help them manage their new professional tasks by providing appropriate information and resources. Our research shows the different approaches of national pharmacists’ associations in using their websites to meet their members’ needs, from discrete intervention to abundant professional materials. Better management of future public health emergencies should result from the experience in dealing with the COVID-19 pandemic. A more coherent approach would need preparedness and response plans adopted by professional associations, with specific good practices for training programs and balanced communication strategies. Those practices could improve the resilience of pharmacists and, therefore, patients, which could save lives.

## References

[1] International Pharmaceutical Federation (FIP). FIP Covid-19 Information Hub (2021). Available from: https://www.fip.org/coronavirus .(Accessed September 18, 2021)

[2] Visacri MB Figueiredo IV Lima TM. Role of Pharmacist during the COVID-19 Pandemic: A Scoping Review. Res Soc Adm Pharm (2020) 17:1799–806. 10.1016/j.sapharm.2020.07.003 PMC7334137 33317760

[3] Airaksinen M Toivo T Jokinen L Savela E Parkkamaki S Sandler C Policy and Vision for Community Pharmacies in Finland: A Roadmap towards Enhanced Integration and Reduced Costs. Pharm Pract (Granada) (2021) 19:2288. 10.18549/PharmPract.2021.1.2288 33628348 PMC7886313

[4] Merks P Jakubowskab M Drelichb E Swieczkowski D Bogusz J Bilmin K The Legal Extension of the Role of Pharmacists in Light of the COVID-19 Global Pandemic. Res Soc Adm Pharm (2021) 17:1807–12. 10.1016/j.sapharm.2020.05.033 PMC7289723 32546449

[5] Koster ES Philbert D Bouvy ML. Impact of the COVID-19 Epidemic on the Provision of Pharmaceutical Care in Community Pharmacies. Res Soc Adm Pharm (2021) 17:2002–4. 10.1016/j.sapharm.2020.07.001 PMC7330552 33317768

[6] Lee L Peterson GM Naunton M Jackson S Bishell M. Protecting the Herd: Why Pharmacists Matter in Mass Vaccination. Pharmacy (2020) 8:199. 10.3390/pharmacy8040199 PMC7712642 33114654

[7] Pritchard RI Huff J Scheinberg N. Impact of Regulatory Changes on Pharmacist-Delivered Telehealth during the COVID-19 Pandemic. J Am Pharm Assoc (2020) 60:76–9. 10.1016/j.japh.2020.06.004 PMC7294253 32593633

[8] Tortajada-Goitia B Morillo-Verdugo R Margusino-Framiñán L Marcos JA Fernández-Llamazares CM. Survey on the Situation of Telepharmacy as Applied to the Outpatient Care in Hospital Pharmacy Departments in Spain during the COVID-19 Pandemic. Farm Hosp (2020) 44:135–40. 10.7399/fh.11527 32646343

[9] Hess K Bach A Won K Seed SM. Community Pharmacists Roles during the COVID-19 Pandemic. J Pharm Pract (2020) 35:469–76. 10.1177/0897190020980626 33317371

[10] Austin Z Gregory P. Resilience in the Time of Pandemic: The Experience of Community Pharmacists during COVID-19. Res Soc Adm Pharm (2021) 17:1867–75. 10.1016/j.sapharm.2020.05.027 PMC7260564 32499160

[11] Turcu-Stiolica A Bogdan M Subtirelu MS Meca AD Taerel AE Iaru I Influence of COVID-19 on Health-Related Quality of Life and the Perception of Being Vaccinated to Prevent COVID-19: an Approach for Community Pharmacists from Romania and Bulgaria. J Clin Med (2021) 10:864. 10.3390/jcm10040864 33669744 PMC7923195

[12] Paul AK Bogart T Schaber AR Cutchins DC Robinson RF. Alaska Pharmacists: First Responders to the Pandemic in the Last Frontier. J Am Pharm Assoc (2021) 61:35–8. 10.1016/j.japh.2020.09.008 PMC7538120 33036935

[13] Dawoud D Chen AMH Rossing CV Garcia-Cardenas V Law AV Aslani P Pharmacy Practice Research Priorities during the COVID-19 Pandemic: Recommendations of a Panel of Experts Convened by FIP Pharmacy Practice Research Special Interest Group. Res Soc Adm Pharm (2021) 17:1903–7. 10.1016/j.sapharm.2020.08.020 PMC7448778 32912829

[14] Toma A Crișan O. Role of Pharmacists’ Associations in Providing Professional Resources during the COVID Pandemic Abstracts of the Annual Meeting of the “Iuliu Hațieganu” University of Medicine and Pharmacy. Med Pharm Rep (2020) 93:S57.

[15] Pharmaceutical Group of the European Union (PGEU). COVID-19 Information Hub (2021). Available from: https://www.pgeu.eu/covid-19-information-hub/ .(Accessed September 18, 2021)

[16] International Pharmaceutical Federation (FIP) FIP Covid-19 Information Hub. Resources from FIP Members and Others (20212). Available from: https://www.fip.org/coronavirus#resources-from-fip-mos .(Accessed September 18, 2021)

[17] Chan AHY Rutter V Ashiru-Oredope D Tuck C Badar ZU. Together We Unite: the Role of the Commonwealth in Achieving Universal Health Coverage through Pharmaceutical Care amidst the COVID-19 Pandemic. J Pharm Pol Pract (2020) 13:13. 10.1186/s40545-020-00214-6 PMC7218554 32426144

[18] Aruru M Truong HA Clark S. Pharmacy Emergency Preparedness and Response (PEPR): a Proposed Framework for Expanding Pharmacy Professionals' Roles and Contributions to Emergency Preparedness and Response during the COVID-19 Pandemic and beyond. Res Soc Adm Pharm (2021) 17:171967–1977. 10.1016/j.sapharm.2020.04.002 PMC7146711 32389631

[19] International Pharmaceutical Federation (FIP). Responding to Disasters: Guidelines for Pharmacy (2016). Available from: https://www.fip.org/files/content/pharmacy-practice/military-emergency-pharmacy/emergency-activities/2016-07-responding-to-disasters-guideline.pdf .(Accessed May 22, 2021)

[20] International Pharmaceutical Federation (FIP). FIP Statement of Policy: Role of the Pharmacist in Disaster Management (2017). Available from: https://www.fip.org/file/1593 .(Accessed September 18, 2021)

[21] International Pharmaceutical Federation (FIP), World Health Organization (WHO). Joint FIP/WHO Guidelines on Good Pharmacy Practice: Standards for Quality of Pharmacy Services, 961. WHO Technical Report Series (2011). 310

[22] OECD. Health at a Glance 2021: OECD Indicators. Paris: OECD Publishing (2021). 273. 10.1787/19991312

[23] OECD/WHO. Improving Healthcare Quality in Europe: Characteristics, Effectiveness and Implementation of Different Strategies. Paris: OECD Publishing/GenevaWHO (2019). p. 447. 10.1787/b11a6e8f-en 31721544

[24] Gaeta M Campanella F Capasso L Schifino GM GentiLe L Banfi G An Overview of Different Health Indicators Used in the European Health Systems. J Prev Med Hyg (2017) 58:E114 28900351 PMC5584080

[25] World Health Organization. Regional Office for Europe. The Legal and Regulatory Framework for Community Pharmacies in the WHO European Region. World Health Organization. Regional Office for Europe (2019).Available from: https://apps.who.int/iris/handle/10665/326394 (Accessed July 25, 2021)

[26] Nguyen E Walker K Adams JL Wadsworth T Robinson R. Reimbursement for Pharmacist-Provided Health Care Services: A Multistate Review. J Am Pharm Assoc (2021) 61:27–32. 10.1016/j.japh.2020.09.009 33069593

[27] Pharmaceutical Society of Australia (2022). COVID-19 information for pharmacists (2021). Available from: https://www.psa.org.au/coronavirus/ .(Accessed July 25, 2021)

[28] Esser F Vliegenthart R. Comparative Research Methods. In: Matthes J General Editor, Davis CS Potter RF, editors. The International Encyclopedia of Communication Research Methods. Hoboken: John Wiley & Sons (2017). 10.1002/9781118901731.iecrm0035

[29] Tight M. Documentary Research in the Social Sciences London: SAGE Publication Ltd (2019). p. 232. 10.4135/9781529716559

[30] National Academies of Sciences, Engineering, and Medicine. Evidence-based Practice for Public Health Emergency Preparedness and Response Washington, DC: The National Academies Press (2020). p. 500. 10.17226/25650 33211452

[31] Finnish Pharmaceutical Association. Current Affairs (2021). Available from: https://www.farmasialiitto.fi .(Accessed September 18, 2021)

[32] Ordre National des Pharmaciens. COVID-19 (2021). Available from: http://www.ordre.pharmacien.fr/Les-pharmaciens/Champs-d-activites/Covid-19 .(Accessed September 18, 2021)

[33] Ordre National des Pharmaciens. Cespharm. Espace Thématique, COVID-19, Documents (2021). Available from: http://www.cespharm.fr/fr/Prevention-sante/Espace-thematique/Covid-19/(result)/doc .(Accessed September 18, 2021)

[34] Hungarian Chamber of Pharmacists. Search Subject COVID-19 (2021). Available from: https://www.mgyk.hu/kereses.html .(Accessed September 18, 2021)

[35] Romanian College of Pharmacists. Search Subject COVID-19 (2021). Available from: https://www.colegfarm.ro/cauta/COVID-19 .(Accessed September 18, 2021)

[36] Consejo General de Colegios Oficiales de Farmacéuticos. Infección por el coronavirus SARS-CoV-2 (COVID-19) (2021). Available from: https://www.portalfarma.com/Profesionales/campanaspf/Asesoramiento-salud-publica/infeccion-coronavirus-2019-nCoV/Paginas/default.aspx .(Accessed September 18, 2021)

[37] American Pharmacists Association. Pharmacists' Guide to Coronavirus (2021). Available from: https://www.pharmacist.com/coronavirus .(Accessed September 18, 2021)

[38] Pharmaceutical Society of Australia. Guidelines for Comprehensive Medication Management Reviews (2021). Available from: https://my.psa.org.au/s/article/guidelines-for-comprehensive-mmr .(Accessed September 18, 2021)

[39] Finnish Pharmaceutical Association. Joint Nordic Declaration to Work for Joint European Vaccine Research and Access (2020). Available from: https://www.farmasialiitto.fi/media/yleiset-tiedostot/joint-nordic-declaration_vaccines_en.pdf .(Accessed September 18, 2021)

[40] Ordre National des Pharmaciens. Cespharm. Covid-19 - PUI: Fiches pratiques - brochure, Covid-19 Fiches pratiques à l’attention des pharmaciens de pharmacie à usage intérieur (2020). Available from: http://www.cespharm.fr/fr/Prevention-sante/Catalogue/Covid-19-PUI-Fiches-pratiques-brochure .(Accessed September 18, 2021)

[41] Hungarian Chamber of Pharmacists. Questionnaire: The Impact of the Covid-19 Epidemic on the Pharmaceutical Profession (2021). Available from: https://www.mgyk.hu/kerdoiv-a-covid-19-jarvany-hatasa-a-gyogyszeresz-szakmara-8230 .(Accessed September 18, 2021)

[42] Romanian College of Pharmacists. Guide on Adapting the Pharmaceutical Practice during COVID-19 Pandemic (2020). Available from: https://www.colegfarm.ro/userfiles/file/ghid%20covid%2029-04-2020.pdf . (Accessed September 18, 2021)

[43] Consejo General de Colegios Oficiales de Farmacéuticos. Información para población general - Otros documentos de interés (2021). Available from: https://www.portalfarma.com/Profesionales/campanaspf/Asesoramiento-salud-publica/infeccion-coronavirus-2019-nCoV/Paginas/informacion-poblacion-general-documentos.aspx .(Accessed September 18, 2021)

[44] American Pharmacists Association. Advocating for You on Coronavirus (2020). Available from: https://aphanet.pharmacist.com/coronavirus/statements-letters .(Accessed September 18, 2021)

[45] Galistiani GF Matuz M Matuszka N Doro P Schvab K Engi Z Determinants of Influenza Vaccine Uptake and Willingness to Be Vaccinated by Pharmacists Among the Active Adult Population in Hungary: a Cross-Sectional Exploratory Study. BMC Public Health (2021) 21:521. 10.1186/s12889-021-10572-8 33731073 PMC7967972

[46] Paudyal V Fialová D Henman MC Hazen A Okuyan B Lutters M Pharmacists’ Involvement in COVID-19 Vaccination across Europe: a Situational Analysis of Current Practice and Policy. Int J Clin Pharm (2021) 43:1139–48. 10.1007/s11096-021-01301-7 34218402 PMC8254632

[47] International Pharmaceutical Federation (FIP). FIP Call to Action to Support Pharmacists and Pharmacy Workers on the coronavirus/COVID-19 Frontline (2020). Available from: https://www.fip.org/files/content/publications/2020/FIP-call-to-action-to-support-pharmacists-and-pharmacy-workers-on-the-coronavirus-COVID-19-frontline.pdf .(Accessed September 18, 2021)

[48] International Pharmaceutical Federation (FIP). An Overview of Current Pharmacy Impact on Immunisation A Global Report 2016. The Hague: International Pharmaceutical Federation (2016). Available from: https://www.fip.org/files/fip/publications/FIP_report_on_Immunisation.pdf (Accessed July 25, 2022)

[49] International Pharmaceutical Federation (FIP). FIP Vaccination Reference Guide: Knowledge and Skills to Support Professional Development and Inform Pharmacy Education in Vaccination The Hague: International Pharmaceutical Federation (2022). Available from: https://www.fip.org/file/5158 .(Accessed July 25, 2022)

[50] Creatis Pharma Software. Pharmacist’s Recommendation (2019). Available from: https://play.google.com/store/ apps/details?id=com.leusoft.recomandareafarmacistului .(Accessed September 18, 2021)

[51] Sociedad Española de Farmacia Hospitalaria. Documento de posicionamiento de la sociedad española de farmacia hospitalaria sobre la telefarmacia (2020). Available from: https://www.sefh.es/bibliotecavirtual/posicionamientos_institucionales/12-POSICIONAMIENTO_TELEFARMACIA_20200510.pdf .(Accessed September 18, 2021)

[52] Chinese Pharmaceutical Association. Coronavirus 2019-nCoV Infection: Expert Consensus on Guidance and Prevention Strategies for Hospital Pharmacists and the Pharmacy Workforce (2020). Available from: https://www.fip.org/files/content/priority-areas/coronavirus/CPA-CORONAVIRUS-2019-nCoV-Expert-Consensus-on-Guidance-and-Prevention.pdf .(Accessed May 27, 2022)

[53] Grech P Grech R. COVID-19 in Malta: The Mental Health Impact. Psychol Trauma (2020) 12:534–5. 10.1037/tra0000925 32478546

[54] Bright B. LiveWell Initiative. LWI COVID-19 Response: Study Protocols for COVID-19 Response in Africa (2020). Available from: https://www.fip.org/files/content/priority-areas/coronavirus/STUDY_PROTOCOLS_FOR_COVID-19_RESPONSE_IN_AFRICA-_an_African_solution_to_the_pandemic_-_Author.pdf .(Accessed May 27, 2022)

[55] Consejo General de Colegios Oficiales de Farmacéuticos. Buenas prácticas en farmacia comunitaria (2018). Available from: https://www.portalfarma.com/Profesionales/Buenas-practicas-profesionales/Paginas/Buenas-practicas-Farmacia-Comunitaria.aspx .(Accessed September 18, 2021)

[56] Canadian Pharmacists Association. Personal Protective Equipment (PPE): Suggested Best Practices for Pharmacies during the COVID-19 Pandemic (2020). Available from: http://www.pharmacists.ca/cpha-ca/assets/File/cpha-on-the-issues/PPE-Best-Practice-Suggestions.pdf .(Accessed May 27, 2022) 10.1177/1715163520963053 PMC7689622 33282020

[57] International Pharmaceutical Federation (FIP). Strategic Plan 2019-2024 (2019). Available from: https://www.fip.org/file/4369 .(Accessed May 22, 2022)

